# Adolescents’ physical activity: cross-national comparisons of levels, distributions and disparities across 52 countries

**DOI:** 10.1186/s12966-019-0897-z

**Published:** 2019-12-30

**Authors:** David Bann, Shaun Scholes, Meg Fluharty, Nikki Shure

**Affiliations:** 1 Centre for Longitudinal Studies, University College London Institute of Education, London, UK; 2 Department of Epidemiology and Public Health, University College London, London, UK; 3 Department of Social Science, University College London Institute of Education, London, UK

**Keywords:** Physical activity, Physical inactivity, Cross-national comparisons, Health inequalities, Adolescent health

## Abstract

**Background:**

Despite global concerns regarding physical inactivity, limited cross-national evidence exists to compare adolescents’ physical activity participation. We analysed data from 52 high- and low-middle income countries, with activity undertaken inside and outside of school in 2015. We investigated gender and socioeconomic disparities, and additionally examined correlations with country-level indices of physical education (PE) curriculum time allocation, wealth, and income inequality.

**Methods:**

We compared adolescents’ reported activity levels inside and outside of school using nationally representative cross-sectional data from 52 high- and low-middle income countries (*N* = 347,935)—the Programme for International Student Assessment (PISA) in 2015. Students reported average attendance (days/week) in PE classes, and the days/week engaged in moderate activity (MPA) and vigorous activity (VPA) outside of school. We also compared gender and socioeconomic disparities, and additionally examined correlations with purported determinants—country-level estimates of PE curriculum time allocation, wealth, and income inequality.

**Results:**

Average activity levels differed substantially both between and within regions, with potentially important differences in distributions identified—such as a bimodal distribution in the U.S. and Canada in PE. Males were more active than females, as were those from households with higher rather than lower household wealth; these disparities were modest for PE, but higher for moderate and vigorous activity outside school—there was strong evidence for heterogeneity in the magnitude of these disparities (e.g., I^2^ > 95% for gender differences across all countries). PE class attendance was positively correlated with PE curriculum time allocation (rho = 0.36); activity outcomes were inconsistently associated with country-level wealth and income inequality.

**Conclusions:**

Our findings reveal extensive cross-country differences in adolescents’ physical activity; in turn, these highlight policy areas that could ultimately improve global adolescent health, such as the incorporation of minimum country-level PE classes, and the targeting of gender and socioeconomic disparities in activity conducted outside of school. Our findings also highlight the utility of educational databases such as PISA for use in global population health research.

## Introduction

Being physically active is widely thought to benefit mental, physical and social health, [1] yet existing evidence suggests a global pandemic of physical inactivity. In 2010, more than 80% of school-going adolescents were estimated to be insufficiently physically active worldwide, [2, 3] yet substantial variation exists between countries [4]. Documenting and understanding these between-country differences is important in order to identify countries and corresponding policies associated with particularly low or high levels of activity and enable benchmarking for future goal setting [5–7].

While there is some evidence that activity levels among adolescents are particularly high in northern European countries, [6, 8] previous cross-country comparisons of adolescent physical activity [6–15] have so far produced limited evidence for a number of reasons. First, cross-national comparisons have been limited in geographic range, being primarily North American/Western European, [7, 10–12, 15] with low- and middle-income countries (LMIC) being under-represented and analysed separately [9, 13, 14]. In several cases, between-country differences in the year of data collection [9, 13, 14, 16] and age [16] may have confounded cross-country comparisons. Second, studies have not analysed activity performed inside and outside of school separately. Since both have different determinants, with modifiable educational policies potentially more important for physical activity undertaken in schools, it is likely to be useful to understand cross-country differences in each component separately.

The analytical strategies employed in previous studies also limits the scope of available evidence. Studies have tended to compare countries using single numerical estimates of activity (e.g. averages or binary prevalence measures)—such comparisons may miss other important differences between countries in the distribution of activity outcomes. Finally, not all studies have compared gender and socioeconomic status (SES) related disparities in activity, which are additional policy concerns, further limiting the available evidence base. This is despite evidence that physical activity levels are higher among males compared with females, [9, 11–13] and are higher amongst those from more socioeconomically advantaged circumstances [11]. Cross-national research enables investigation of the correlates and determinants of physical activity which operate at the country level. Factors such as country-level economic development and income inequality are noted in highly cited papers as being crucial determinants of adolescent health [5] yet to date have been inconsistently associated with cross-national differences in adolescent physical activity levels in previous studies [6, 7].

Using a large-scale education achievement database to our knowledge previously unused in the epidemiological literature—the Programme for International Student Assessment (PISA)—we compared adolescent physical activity levels across 52 countries measured in 2015. This dataset spans both high- and low-income countries, and activities undertaken both inside and outside of school. Three primary outcomes were used: days/week and time spent in physical education (PE) classes, and days/week engaged in moderate (MPA) and vigorous (VPA) activity outside of school. Since single numerical estimates may mask other meaningful cross-national differences, we also compared the distribution of each activity outcome, and additionally investigated gender and SES disparities. Finally, we additionally examined whether country-level PE curriculum time allocation was correlated with the PISA assessed levels of PE, and examined whether two structural factors thought to be key determinants of adolescent health [5] —national levels of wealth and income inequality—were correlated with levels of activity both inside and outside of school.

## Methods

### Data source

PISA is conducted by the Organisation for Economic Co-operation and Development (OECD) in over 70 member and non-member nations and economies [17]. PISA samples pupils in each country aged between 15 years and 3-months and 16 years and 2-months at the time of assessment. PISA has a two-stage probabilistic, stratified and clustered survey design. First, schools are stratified and then randomly selected with probability proportional to size (a minimum of 150 schools was selected from within each country). All countries and economies must ensure they meet the OECD’s response rate of 85% for schools and 80% for pupils in order to be included in the study; Malaysia was not included in PISA 2015 as a result. PISA is designed to obtain representative samples of the in-school population of adolescents in each participating country; as such, it does not necessarily represent the population of adolescents who do not attend school. Further details of the sampling protocols are shown in the 2015 Technical Report [17]. PISA has taken place every 3 years since 2000 yet physical activity data were included only in 2015.

Over half a million students participated in 2015, representing about 29 million students in the schools of the 72 participating countries and economies. To aid comparison, we restricted our analyses to 52 countries with available physical activity data: additional sub samples (‘economies’) were not included given concerns about national representation (e.g., the only four regions sampled in China were Beijing, Shanghai, Jiangsu, and Guangdong). To facilitate comparisons with previous and future work, we grouped countries into six categories based largely on the World Health Organization (WHO) regions and sub-regions. Sub-regions are denoted by A, B and C suffices, which typically map onto declining country-level wealth (for example, see https://www.who.int/choice/demography/euro_region/en/). The six categories were as follows: (1) Americas A (U.S. and Canada); (2) Americas B/C; (3) Eastern Mediterranean; (4) Europe A (e.g., the United Kingdom and other Western European nations); (5) Europe B/C (e.g., Poland and Hungary); and (6) South-East Asian & Western Pacific (hereafter referred to as SE Asian + Pacific). We combined the SE Asian and Pacific Regions as Thailand was the only PISA participant in the former. We grouped Chinese Taipei and Hong Kong into the SE Asian + Pacific Region based on geography even though they are not currently recognized WHO member states. Montenegro was grouped into the Europe B/C category for the same reason. Further details of the PISA 2015 study are available elsewhere [17].

### Physical activity

Students were asked to report *outside of school* the number of days during which they engaged in moderate physical activity (hereafter referred to as MPA: such as walking, climbing stairs or riding a bike to school) for at least 60 min per day during the week before the PISA assessment. A similar question was asked for vigorous activity (hereafter referred to as VPA: such as running, cycling, aerobics, soccer and skating that makes you sweat and breathe hard) for at least 20 min/day. PISA also asked students, on average, on how many days they attended PE classes *during school* each week throughout the school year. Each outcome was summarised as days/week (range: 0–7).

### Socioeconomic status (SES)

SES was measured by reported family wealth possessions, a continuous variable estimated using item response theory scaling by the OECD. We calculated SES using eight standardised questions on possessions in and characteristics of the home. These included questions on whether the home has an internet connection, whether the student has her own room, the number of rooms in the home with a bath or shower, the number of televisions, computers, tablets, and e-book readers in the home, the number of cars the family has, and three country-specific wealth items [17]. Country-specific quintiles of this continuous variable were calculated for use in our analyses.

### Statistical analysis

For each country, we calculated the mean number of days that students: (1) attended PE classes each week during the school year; (2) engaged in MPA in the last week (for ≥60 min/day) outside of school; and (3) engaged in VPA in the last week (for ≥20 min/day) outside of school. To account for potential non-normality of outcome data—at the potential expense of loss of outcome variance and thus information—analyses were also conducted using binary outcomes: the proportion of adolescents who engaged in activity for five or more days/week (MPA and VPA) and the proportion who took part in PE classes for three or more days/week (due to its lower levels). Time spent in PE classes was calculated by multiplying the number of PE classes per week by the reported average class time. Using data aggregated at the country level, Spearman (rho) and Pearson correlational analyses were performed to verify the PISA data by comparing each indicator to the WHO 2010 compiled estimates of insufficient physical activity among school-going adolescents (aged 11–17 years) of both genders (% < 60 min/day of moderate- to vigorous-intensity activity) [18]. Students with missing data for gender, SES, and physical activity were excluded from analyses. To inform the potential for this in biasing our findings, logistic regression was used to examine demographic differences between students with and without physical activity data.

Cross-national comparisons were made by estimating mean (95% CI) activity levels within each country (days/week); these were calculated separately by gender and SES (top versus bottom wealth quintile given evidence for linearity) to examine disparities. We decided, a priori, to calculate wealth quintile specific estimates separately for male and female students due to expected gender differences as reported in the literature [4, 13]. Meta analyses were used to formally test heterogeneity in the gender differences—both within- and between-regions—using the I^2^ statistic to quantify the percentage of variation across nations due to heterogeneity rather than chance [19]. These analyses were repeated for differences by wealth quintile. Comparisons between countries’ physical activity distributions (e.g. the percentage of students active on 0, 1 and 2 days/week) were made by both tabulating and plotting via histograms.

Additional ecological analyses were conducted to examine factors plausibly correlated with—or be structural determinants of—cross-national differences in mean levels of activity. First, we examined correlation coefficients between PE class attendance and country-level PE curriculum time allocation for secondary schools (mean minutes/week) estimated by the United Nations Educational, Scientific and Cultural Organization (UNESCO) in 2014 [20]. Second, we examined correlation coefficients between all activity outcomes and two economic outcomes collated by the World Bank—national wealth (as indexed by gross domestic product (GDP) per capita) and income inequality (as indexed by the Gini coefficient) in 2015 (or nearest neighbouring year to 2010 if not available in 2015) [21]. In addition, we examined the gender-specific correlation coefficients between estimated SES disparities (top-wealth quintile minus bottom-wealth quintile) and income inequality.

Analyses were performed using Stata V15.0 following the recommended use of the Balanced-Repeated-Replication (BRR) weights (final student response and replicate weights) to account for the amount of uncertainty due to sampling error, including the clustering of students within schools [22]. Analytical syntax and accompanying datasets to enable replication of our findings are available at https://github.com/dbann/pisa.

## Results

Sample characteristics and descriptive statistics are summarised in Additional file 1: Table S1. Data on physical activity by gender was available for *N* = 347,935 students, across 52 countries with an average (median) sample size of 5557 (range: 3150–18,680); Additional file 1: Figure S1 shows a flow diagram. Physical activity data was missing for 37,696 students; of those with PA data, information on SES was missing for 1489 students. Lower family wealth, lower parental education, and being male were associated with increased odds of having missing data (*P* < 0.001 in all cases; data not shown). At the country level each PISA assessed activity outcome was inversely correlated with the WHO 2010 prevalence estimates of insufficient physical activity (PE classes: Spearman’s rho = − 0.09; MPA: rho = − 0.24; VPA: rho = − 0.24; Additional file 1: Table S2). Mean, median, and the distribution of all activity outcomes are shown in Additional file 1: Table S3.

### Country differences in physical activity

**Fig. 1:**
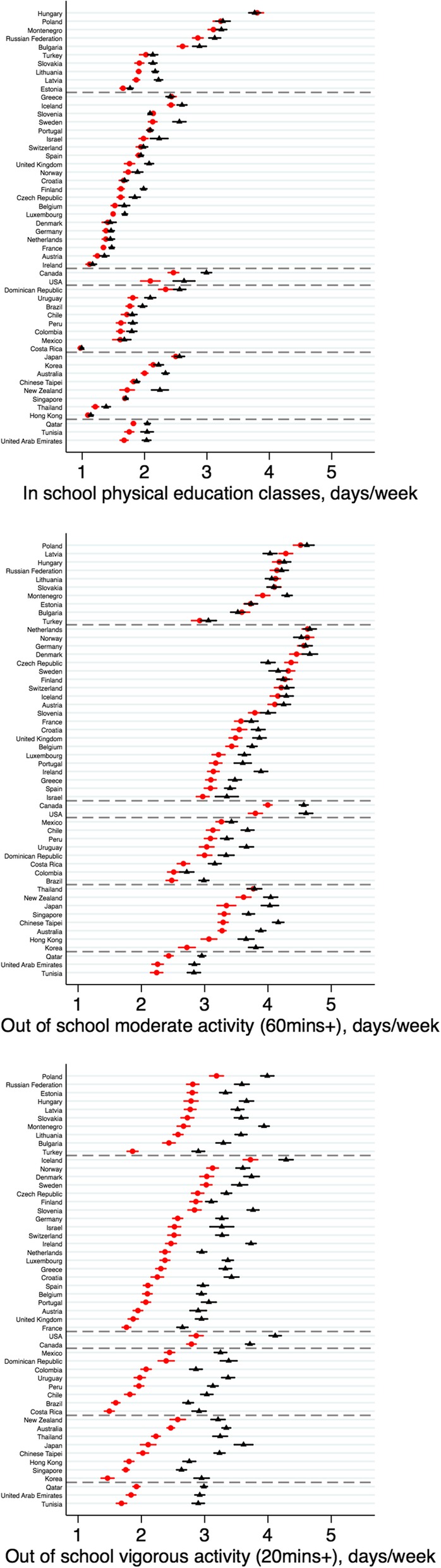
Gender disparities in adolescents’ mean (95% CI) physical activity: in school and out of school. Note: females = red circles; males = black triangles

There were large differences between regions in participation: activity levels inside and outside of school were typically highest in Europe B/C nations; activity levels outside of school were lowest in the Eastern Mediterranean region (Fig. 1 shows mean differences; Additional file 1: Figure S2 shows binary prevalence differences). There were also notable differences within regions. For example, within Europe B/C, average days/week in PE classes in Hungary were approximately double those in Estonia among both genders, whilst moderate activity levels outside of school were higher by approximately 0.5 days/week.

**Fig. 2:**
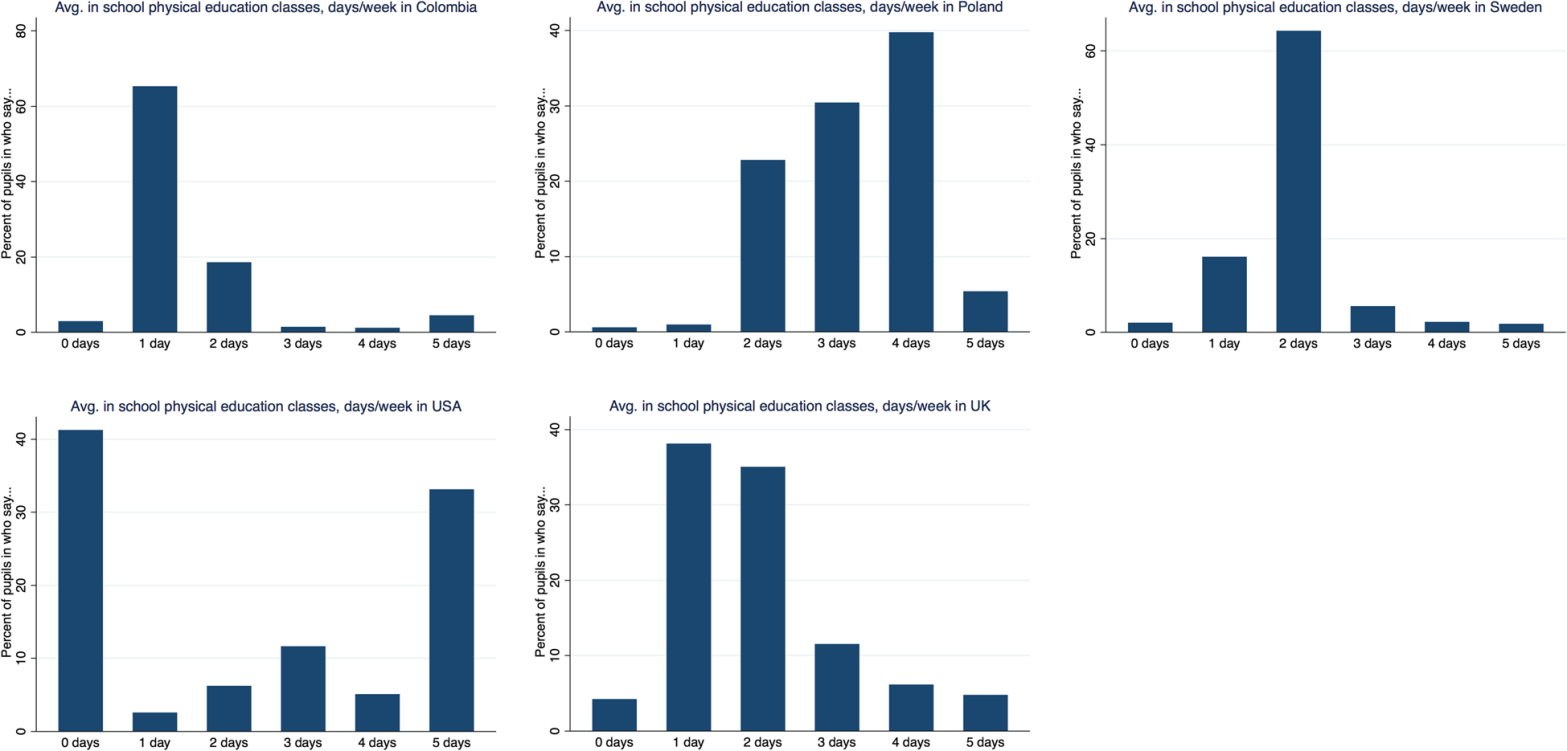
Histograms showing distributions of in-school physical activity participation in five countries with differing distributions. Note: Colombia (mean 1.7 days/week; 1.7 h/week), Poland (mean 3.2 days/week; 2.8 h/week), Sweden (mean 2.3 days/week; 2.4 h/week), U.S. (mean 2.4 days/week, 2.3 h/week), UK (mean 1.9 days/week; 2.0 h/week)

There was substantial diversity in the distribution of activity outcomes, particularly for activity inside school, revealing cross-country differences not found when using a single numerical summary measure—either mean or prevalence estimates (Fig. 2; Additional file 1: Table S4). For example, activity levels inside school in the U.S. showed a bimodal distribution (mean PE class attendance 2.3 days/week; 41.3, 6.3 and 33.1% of students attended PE classes on 0, 2 and 5 days/week respectively), as did those in Canada. In contrast, most other countries exhibited more centrally shaped distributions (e.g. Sweden: mean 2.3 days/week; 2.0, 64.3 and 1.8% of students attended PE classes on 0, 2 and 5 days/week respectively).

### Gender disparities

Males were more active than females, for activities inside and outside of school (Fig. 1). Gender disparities however were most pronounced for activities outside of school and were larger for VPA than for MPA—the average days/week spent in VPA was 3.4 among males and 2.3 among females; for MPA, the values were 3.9 and 3.4 days/week, respectively. Gender disparities were also found using the binary outcomes (Additional file 1: Figure S2; Additional file 1: Table S5 shows distributions across all activity categories). For each outcome, there was strong evidence for heterogeneity in the magnitude of gender differences—across all countries (I^2^ > 95% for all outcomes, *P* < 0.001)—as well as between- and within-regions (see Additional file 1: Figure S3 for forest plots and heterogeneity test statistics).

Cross-national differences in gender disparities was most pronounced for VPA: being highest in the Americas B/C and Eastern Mediterranean. However, there was evidence for heterogeneity within each region—for example in Europe A, which had lower gender disparities than other regions, differences ranged from 1.26 (95% CI: 1.15, 1.38) days/week higher activity among males vs females in Ireland, to 0.24 (0.14, 0.35) in Finland. In some countries, gender disparities in average levels reflected differences at the upper tail of the distribution. For example, the average days/week engaged in MPA was 3.9 for males and 3.3 for females in Australia; 23.9% of males engaged in MPA 7 days a week, while 13.2% of females did so.

### SES disparities

**Fig. 3:**
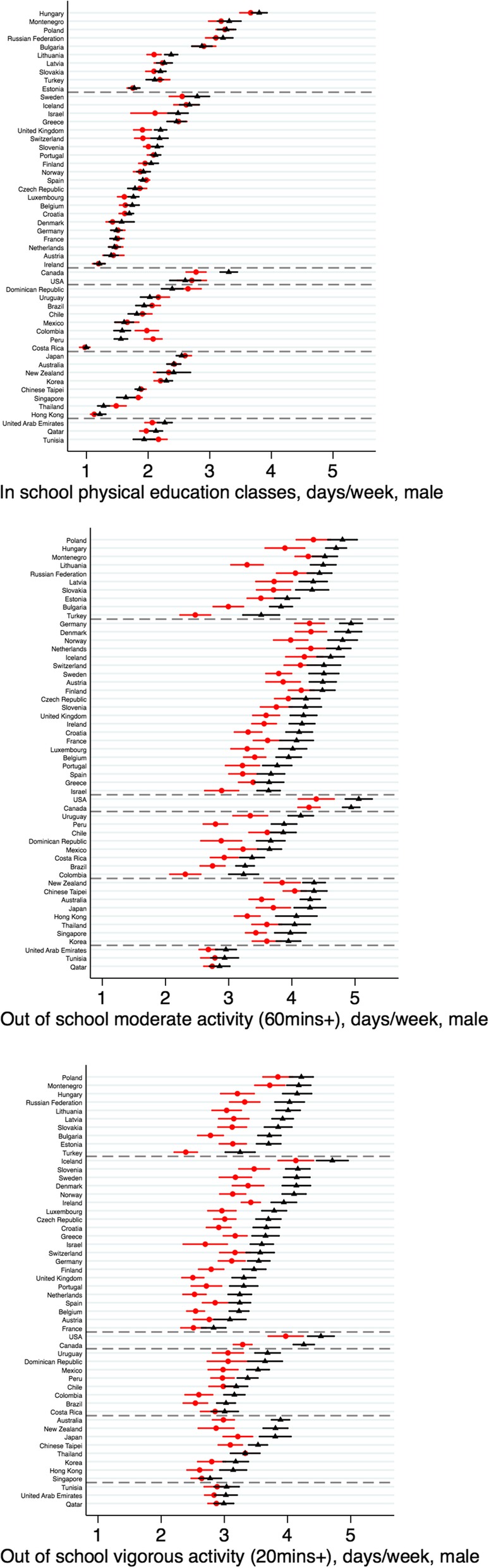
Socioeconomic (wealth-based) disparities in adolescents’ mean (95% CI) physical activity: in school and out of school among males. Note: lowest quintile = red circles; highest quintile = black triangles

**Fig. 4:**
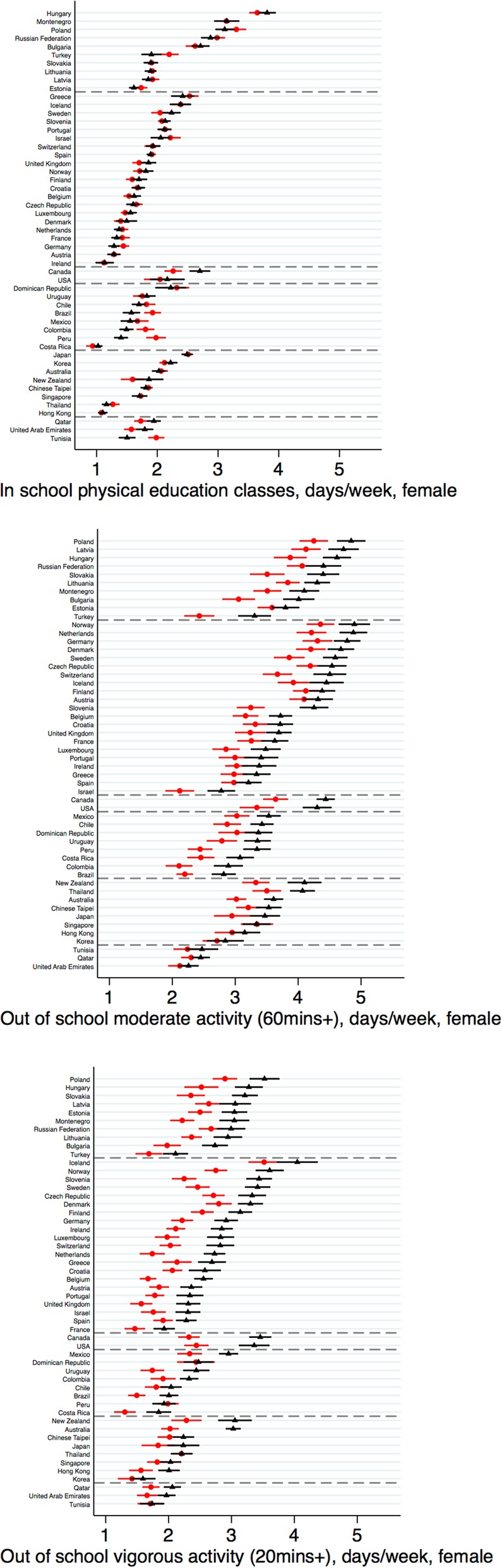
Socioeconomic (wealth-based) disparities in adolescents’ mean (95% CI) physical activity: in school and out of school among females. Note: lowest quintile = red circles; highest quintile = black triangles

SES disparities were largest for activities outside of school (Figs. 3, 4 show the averages for males and females respectively; Additional file 1: Table S6 shows the distributions). For both genders, activity levels for MPA and VPA were on average 0.5 to 0.6 days/week higher for students in the top- versus bottom-wealth quintile; these wealth disparities were also found using the binary outcomes (Additional file 1: Figures S4–S5). Regional variation was lower for SES than for gender disparities (I^2^ > 67% across outcomes; forest plots are shown in Additional file 1: Figures S6–S7). The gap between the wealth quintiles was particularly high among females for VPA in the Americas A (e.g. 1.13 (1.00, 1.27) days/week higher for females in the top- versus bottom-quintile in Canada). SES disparities in VPA were notably lower in the Eastern Mediterranean region. For in-school activity, regional estimates differed in direction (e.g., higher activity in the top quintile in Americas B/C among females compared with higher activity in the bottom quintile among females in Americas A).

### Ecological analyses

**Fig. 5:**
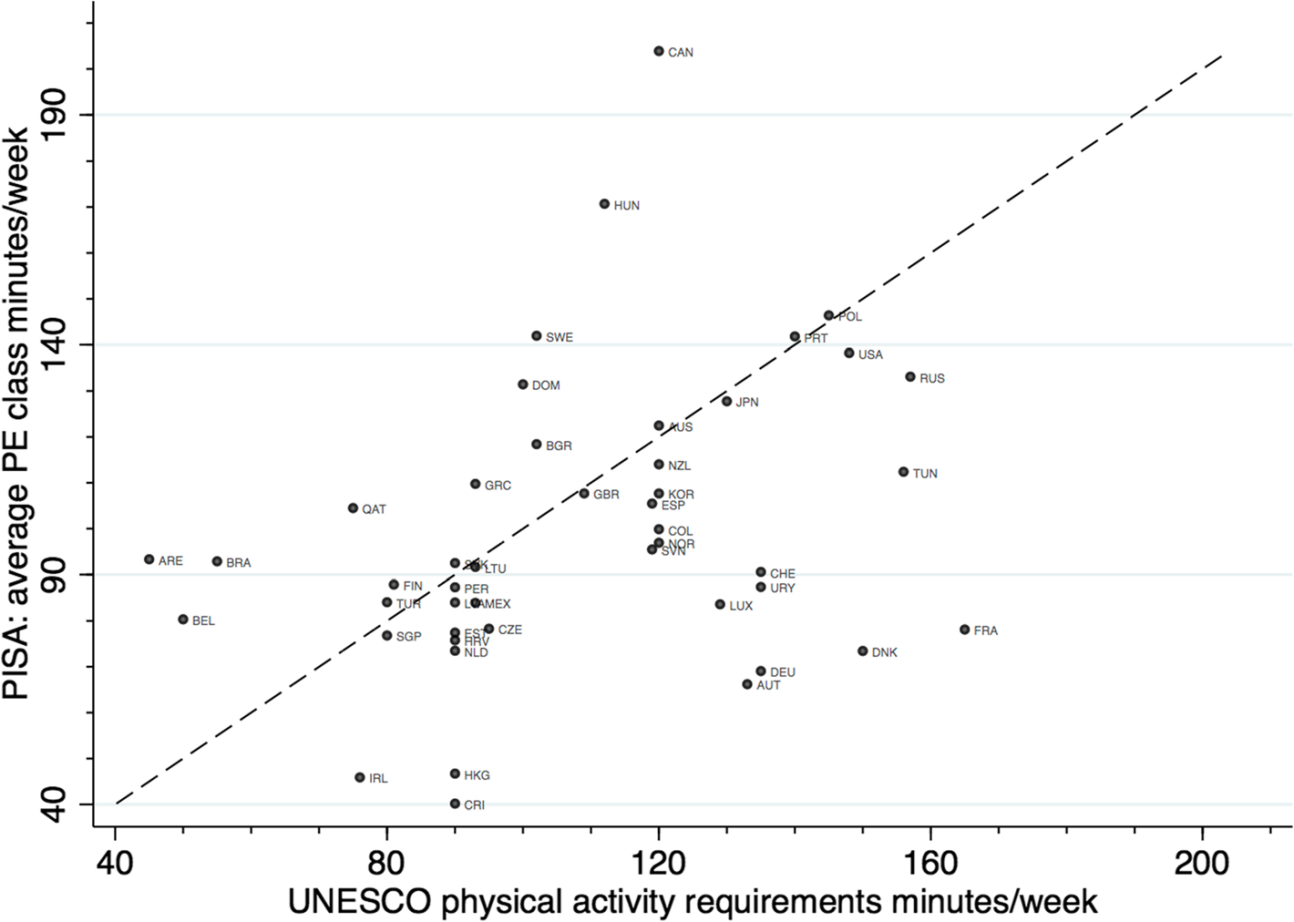
Scatter plot between country-level physical education (PE) curriculum time allocation per week and average in school physical activity minutes per week. Note: Graph includes 45-degree line

**Fig. 6:**
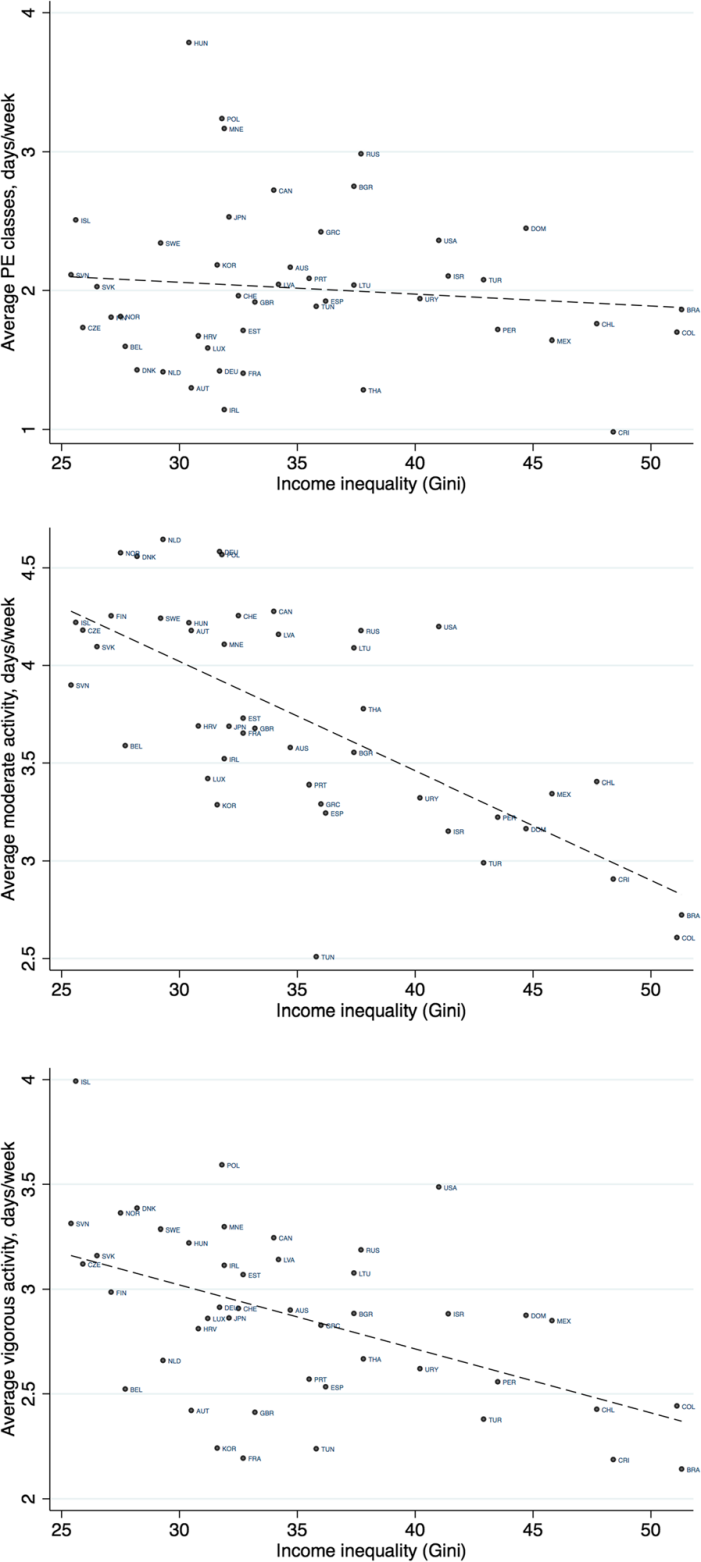
Scatter plots between country-level income inequality (Gini coefficient) and average physical activity levels. Note: lines of best fit are shown. Higher values of the Gini coefficient indicate higher income inequality

Figures 5, 6 and Additional file 1: Tables S2 and 7 show the results of the ecological analyses. Country-level PE curriculum time allocation for secondary schools (mean minutes/week) were positively correlated with the PISA assessed levels of activity inside school (PE class attendance: rho = 0.26; number of days multiplied by average class time: rho = 0.36). National wealth as indexed by GDP was weakly negatively correlated with levels of activity inside school (rho = − 0.14), yet positively correlated with activity outside of school (MPA: rho = 0.40, VPA: rho = 0.21). Income inequality as indexed by the Gini coefficient was negatively correlated with levels of activity outside of school (MPA: rho = − 0.67, VPA: rho = − 0.48) yet not with levels of activity inside school (rho = 0.01). Income inequality was also negatively associated with the SES disparities in activity in school and in vigorous activity outside school—countries with more unequal income distributions tended to have lower SES disparities in these physical activity outcomes (Additional file 1: Table S7). Findings were similar using either mean activity levels or binary outcomes.

## Discussion

Using a large-scale global education database, we identified substantial cross-country differences in adolescents’ physical activity. Our findings extend those conducted in either high [7, 12, 15] or low-middle [13] income countries by including a greater number of countries across income levels, using more recent data (2015), and expanding on the comparisons across countries. We examined activities conducted inside and outside of school separately, compared the distributions of activity in addition to averages, quantified gender and SES disparities, and examined correlations with national-level economic factors thought to be key determinants of adolescent health.

There are several explanations for cross-country differences in adolescents’ physical activity, which if confirmed, should lead to multiple avenues for policy development. For activity conducted inside school, we anticipated that cross-country differences in laws or guidelines mandating PE class participation are likely to be a main source of variation. Our analyses partly support this suggestion, given the positive (albeit weak-moderate) correlation between the PISA assessed indicator (days/week attending PE classes throughout the school year) and the UNESCO compiled estimates of PE curriculum time allocation in secondary schools. For example, Hungary has reportedly higher levels of PE time allocation in secondary schools than Estonia (145 vs 90 min/week), consistent with our findings for these countries which showed the average days/week in PE classes to be twice as high. The large heterogeneity within the U.S. revealed by the UNESCO study (e.g., 30 min/week in Iowa versus 200 min/week in California), [20] which we were not powered to investigate, is potentially partly reflected in our own finding in the U.S. PISA sample of a bimodal distribution for activity in school (shown in Fig. 2). Consistent with our findings, a recent nation-wide study in the U.S. also found a bimodal distribution which persisted from 1991 to 2015: possibly reflecting the fewer opportunities for PE in high-poverty schools [23]. Discrepancies between PE time allocation and observed levels of activity participation in schools in many countries, all those below the 45 degree line in Fig. 5, may suggest that laws or guidelines are not being implemented sufficiently, thereby requiring action to redress. For example, Denmark’s 2016 Report Card on Physical Activity for Children and Youth suggested that high investment and government-led initiatives to support physical activity have not translated into higher observed physical activity levels [24]. Other education policies which could explain country differences in activity include whether PE class length is enforced with mandatory minimum of minutes (e.g., 135 min/week in Poland, [25] yet no mandatory minimums exist across England, Colombia, nor all of the U.S.); the funding, availability and quality of facilities within schools; and the training of PE teachers [6]. The importance of these factors on cross-national differences in activity participation inside schools warrants future empirical investigation, yet is likely to be challenging given lack of consistent data across countries, [6] and the possibility of reverse causality (since education policies may arise due to concerns about prior low physical activity levels which track across time). In addition to educational policies (and their implementation), other plausible explanations for differences between countries include social norms or cultural differences regarding the value of PE, particularly if time spent in PE classes is interpreted as being in competition with academic achievement [26].

The cross-country differences in physical activity levels outside of school shown in our study are likely influenced by a greater range of determinants operating through several levels of influence (i.e. individual, social and built environmental, and policy). These include economic factors which partly determine the resources and the quality of the environments which facilitate participation, including the opportunities available for active transportation to and from school, and cultural factors, such as country-level beliefs regarding the importance of physical activity for health and personal/group identity. In support of the potential importance of non-economic factors, there was little evidence in our study of correlation between in-school physical activity and GDP. Such cultural factors are difficult to measure but may be potentially fruitful targets for identification and modification to increase activity levels. Cultural factors may also partly explain the (pro-male) gender disparities in physical activity outside school that we observed, which were largest for VPA. However, it is unclear which specific factors explain these disparities [27]. For example, a recent systematic review found weak evidence that social support was associated with physical activity levels among female adolescents—yet the majority of studies were conducted in one country (the U.S.), most were cross-sectional (81%), and the authors noted a high risk of selection bias in the included studies [28].

Factors such as country-level economic development and income inequality are noted in highly cited papers as being crucial determinants of adolescent health [5] yet to date have been inconsistently associated with cross-national differences in adolescent physical activity levels in previous studies [6, 7]. Our findings add to this evidence base. While we observed that national levels of income inequality strongly negatively correlated with levels of activity outside of school (especially vigorous activity), it is unclear why this is the case. It remains speculative as to whether national levels of income inequality has a causal effect on activity participation (and if so, what factors mediate this effect) or if there are other factors such as those related to economic development—changing patterns of transportation, increased use of technology and urbanization [29]—which operate in such a way to result in a spurious association [30].

As with all physical activity measures, there is uncertainty in the extent to which these self-reported measures provide unbiased, reliable estimates of long-term physical activity levels. Systematic differences in over- or under-reporting activity may bias differences between countries, as well as country-differences in gender and SES disparities. Empirical validation of these measures may therefore be beneficial, notwithstanding challenges in identifying a tractable gold-standard comparison given limitations in device-assessed physical activity measures (e.g., typically shorter time spans of investigation, higher non-response rates, and potential bias due to the Hawthorne effect). Nevertheless, combining self-report and device-measured activity may be useful in future comparisons. Reassuringly, the pro-male gender disparities we observed are also found in studies which utilise device-assessed physical activity [12].

While our study included more countries than previous studies, inclusion of other countries would expand the possibilities for cross-country comparison; these include low-middle income countries such as mainland China and India, which account for large fractions of the total adolescent population worldwide. We also included multiple activity outcomes, asked in identical form in each country; these enabled international comparisons of activity participation both inside and outside of school. Each outcome correlated in the expected direction with activity data aggregated by the WHO, [18] despite measurement differences likely weakening such correlation (e.g., exact ages sampled, scale of measurement, and year of data collection). In addition, more detailed reported information on PE is likely to be beneficial to include in other comparative studies given its multiple potential consequences beyond simply increasing short-term activity levels—such as in facilitating (or at least not impairing) academic achievement in other subjects, and in promoting future (potentially lifelong) physical activity participation.

While the sample framework for PISA is designed to enable national representation of the in-school population of adolescents, as in all cross-country comparisons there may be between-country differences in unobserved factors which could confound our findings (e.g., differences in sample selection within each country, or time of year of measurement); as such, triangulation from other data sources may be useful. Further research within and between countries is also needed to examine the extent to which cross-country differences in adolescent physical activity (and their determinants) are different to those in other key life stages—childhood, adulthood, and older adult life.

In summary, our findings suggest substantial variation in adolescents’ activity across regions and countries. The presence of these differences suggests that the global pandemic of physical inactivity is not universal—it may be averted by understanding and adopting global best-practices. This may include increasing guidelines for physical activity within schools, particularly since several large physical activity interventions focusing on knowledge and motivation within schools have yielded null findings [31, 32]. Future cross-national comparative research may benefit from 1) extending the physical activity outcomes to include those undertaken both inside and outside of school; 2) triangulating across different data sources to increase the robustness of the estimated physical activity levels observed in each country; and 3) investigating the modifiable factors which explain such between-country differences—including differences in the average levels, distributions, and disparities in physical activity.

## Supplementary information

**Additional file 1: Table S1.** Descriptive statistics for the countries included in the PISA sample. **Table S2.** Correlations between country-level physical activity outcomes and country-level factors. **Table S3.** Mean, Median, and SD in physical activity outcomes by country. **Table S4.** Frequencies of physical activity outcomes by country. **Table S5.** Tabulations of physical activity outcomes by gender. **Table S6.** Tabulations of physical activity outcomes by wealth (top and bottom quintile). **Table S7.** Correlations between socioeconomic disparities in physical activity and income inequality. **Figure S1.** A flow chart showing derivation of the analytical sample. **Figure S2.** Gender disparities in adolescents’ (95% CI) physical activity: in school and out of school. **Figure S3A.** Forest plot of gender differences in physical activity outcomes (vigorous activity outside of school). **Figure S3B.** Forest plot of gender differences in physical activity outcomes (moderate activity outside of school). **Figure S3C:** Forest plot of gender differences in physical activity outcomes (in school activity). **Figure S4.** Socioeconomic (wealth-based) disparities in adolescents’ (95% CI) physical activity: in school and out of school among males. **Figure S5.** Socioeconomic (wealth-based) disparities in adolescents’ (95% CI) physical activity: in school and out of school among females. **Figure S6A.** Forest plot of socioeconomic (wealth) differences in physical activity outcomes (vigorous activity outside of school), among males. **Figure S6B.** Forest plot of socioeconomic (wealth) differences in physical activity outcomes (moderate activity outside of school), among males. **Figure S6C.** Forest plot of socioeconomic (wealth) differences in physical activity outcomes (in school activity), among males. **Figure S7A.** Forest plot of socioeconomic (wealth) differences in physical activity outcomes (vigorous activity outside of school), among females. **Figure S7B.** Forest plot of socioeconomic (wealth) differences in physical activity out-comes (moderate activity outside of school), among females. **Figure S7C.** Forest plot of socioeconomic (wealth) differences in physical activity outcomes (in school activity), among females.

## Data Availability

The datasets supporting the conclusions of this article are available from the OECD http://www.oecd.org/pisa/data Analytical syntax and other accompanying datasets to enable replication of our findings are available at https://github.com/dbann/pisa Other datasets used in this paper include economic data (freely available from the World Bank: https://data.worldbank.org/ and via the Stata command *wbopendata*) and data collated by the UNESCO (source: unesdoc.unesco.org/images/0022/002293/229335e.pdf; derived variables: https://github.com/dbann/pisa).
